# The ecology of medical care on an isolated island in Okinawa, Japan: a retrospective open cohort study

**DOI:** 10.1186/s12913-017-1979-8

**Published:** 2017-01-14

**Authors:** Makoto Kaneko, Masato Matsushima, Greg Irving

**Affiliations:** 1 Musashikoganei Clinic, Japanese Health and Welfare Co-operative Federation, 1-15-9 Honcho, Koganei, Tokyo, 184-0004 Japan; 2 Division of Clinical Epidemiology, Jikei University School of Medicine, 3-25-8 Nishishimbashi, Minato-ku, Tokyo, 105-8461 Japan; 3 Institute of Public Health, University of Cambridge, Cambridge, CB23 OSR UK

**Keywords:** Primary health care, Ecology, Medical care, Physician visit, General practice

## Abstract

**Background:**

We aimed to describe the ecology of medical care on an isolated island with limited access to secondary care, and to evaluate the gatekeeping function of the island’s primary care clinic through comparison with a previous nationwide survey.

**Methods:**

We conducted this retrospective, open cohort study on Iheya, an isolated island in Okinawa Prefecture that has one primary care clinic. We considered Iheya as unique location in which to examine the role of primary care in Japan. Participants were patients who visited the island’s clinic between February 1, 2013 and January 31, 2014. We calculated the number of visits to the clinic and referrals to off-island medical facilities using electronic medical records. We also compared data for Iheya with a nationwide survey conducted in 2003.

**Results:**

Iheya had 1314 inhabitants in 2013. Of the 5682 visits to the clinic in the 1-year study period, 290 people were referred to off-island medical institutions. There were 64 referrals to emergency departments; of these, 57 people were admitted to hospital. The rate of visits to the clinic per month per 1000 inhabitants was 360.4 visits (95% confidence interval: 351.0–369.7). Of these, 18.4 (16.3–20.5) were referred off-island, with 4.1 (3.1–5.1) referrals to emergency departments and 3.6 (2.6–4.6) hospitalizations. Despite the high incidence of visits to the primary care clinic, the rates of hospital-based outpatient clinic visits, emergency department visits, and hospitalizations were lower than rates reported in a previous Japanese study.

**Conclusions:**

This suggests that several dimensions of primary care, its gatekeeping function in particular, are likely to play important roles in this geographical setting.

## Background

In 1961, White et al. [1] introduced “the ecology of medical care” or healthcare-seeking behavior per 1000 people in the general population over a 1-month period, as a way to assess the adequacy of medical resources. Although this model is widely referenced by policy makers and educators [2–9], an ecological model is likely to vary given differences in populations, regional areas, and times [10]. In 2005, Fukui et al. [11] reported the ecology of medical care as basic data for healthcare-seeking behavior in Japan using 2003 questionnaire and health diary data; unfortunately, that study did not include referrals data. Referral is an important primary care function, as several dimensions of primary care, particularly access, comprehensiveness, and coordination of primary care services, reduce the number of visits to specialists and emergency departments [12].

Japan has the fastest-growing super-aging population of most developed countries [13]. In 2013, the Japan Ministry of Health, Labour and Welfare highlighted the importance of primary care in managing the aging population [14]. Unfortunately, it is difficult to evaluate the role of primary care in Japan using the ecology of medical care model, as Japan has a “free access system” that guarantees access to all medical facilities. Therefore, people can bypass primary care clinics and visit secondary or tertiary medical services directly. This is notable in urban areas with more medical services. In contrast, the ecology of medical care is easy to evaluate on an isolated island where only primary care is available.

Therefore, we assessed healthcare-seeking behavior on Iheya, an isolated island in Okinawa, Japan. The island has one primary care clinic, one dental office, and one nursing home. There are two public health nurses in the village office, but there are no nurse practitioners/physician assistants/nurse midwives, alternative/complementary medicine facilities, or medical specialists (e.g., optometrists/podiatrists), and the island does not have a pharmacy. Neither surgical facilities nor hospitals with beds are available, and patients requiring advanced care are referred to off-island secondary facilities [15]. A helicopter service is available to transport patients to the main island of Okinawa in medical emergencies.

The present study aimed to describe the ecology of medical care on Iheya, an isolated island where access to secondary care service is limited, and to evaluate the gatekeeping function of the primary care clinic, based on comparison with a previous nationwide survey.

## Methods

**Fig. 1:**
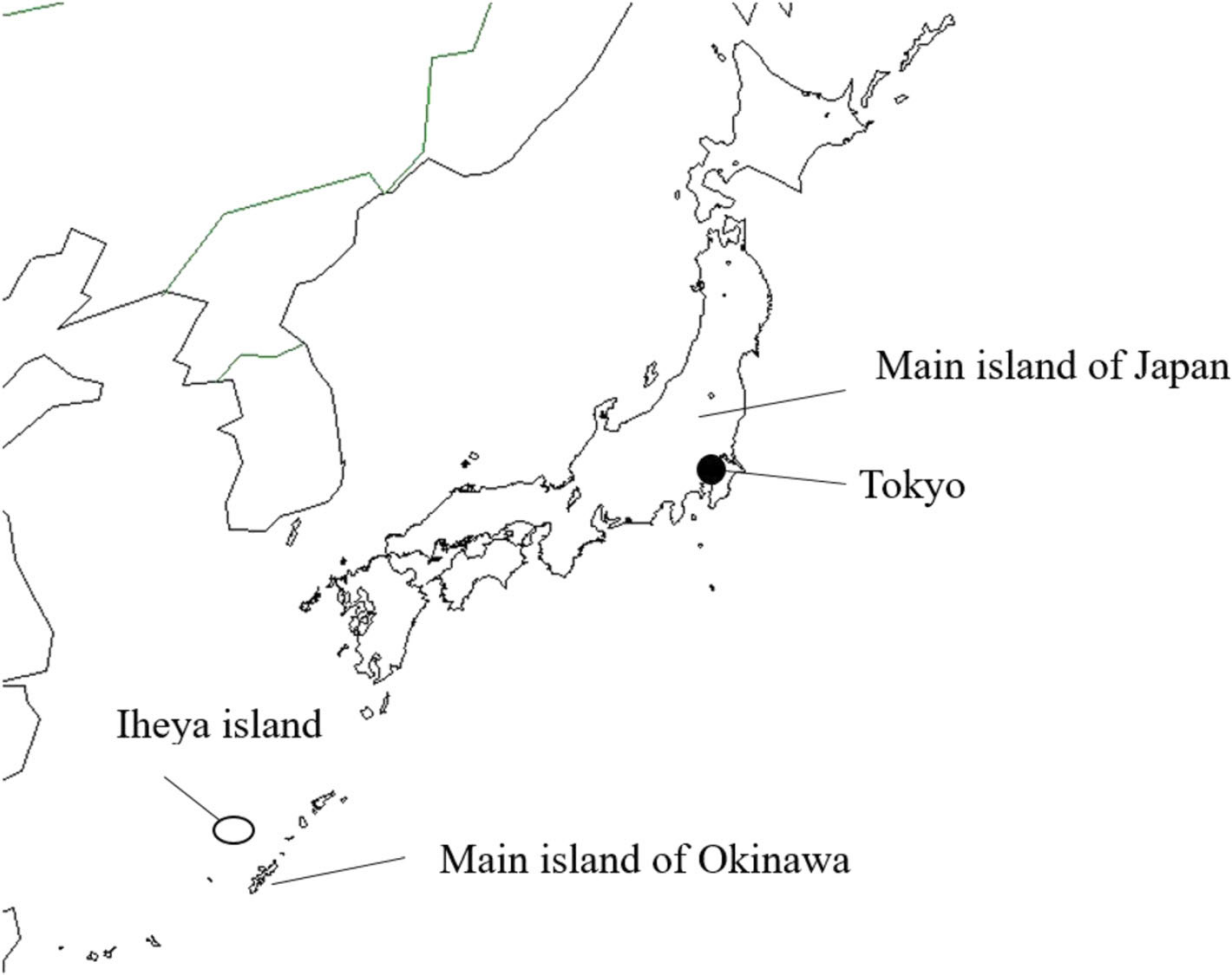
Geographical location of Iheya island in relation to mainland Japan

We conducted a retrospective, open cohort study on Iheya, an isolated island in Okinawa Prefecture, Japan (Fig. 1). The island’s area is 20.59 km^2^ and its circumference is 34.23 km. The island is located 41.1 km from the main island of Okinawa (90 min by ship: one-way fare approximately 2440 yen). Iheya had 1314 inhabitants as at January 31, 2013.

One of the present authors (MK) worked as a physician on Iheya from April 1, 2011 to March 31, 2014. The clinic had four staff members (one physician, one nurse, and two clerks), but no beds. Emergency medical services were available 24 h per day, and the clinic received approximately 30 emergency calls per month. The clinic had a microscope, electrocardiogram, X-ray and ultrasound equipment, and the capacity to conduct simple blood tests.

### Participants

All patients who visited the island’s clinic between February 1, 2013 and January 31, 2014 were included in the present study. There were 1314 inhabitants in total (male/female: 679/635), and 25.1% of the island’s population was aged 65 years and over (Okinawa Prefecture, 17.0%; Japan overall, 23.4%) and the ratio of the population aged under 15 years was 18.3% (Okinawa Prefecture, 17.7%; Japan overall, 13.2%) [16]. During the study period, 79 people moved off the island, and 83 people moved to the island. The population at the start and end of the study was almost the same. Therefore, we used the population at the start of the study as the denominator for each proportion.

### Main outcome measures

We retrospectively counted the number of patient visits to the clinic and referrals to off-island medical facilities by examining the electronic medical record system (Dr. Bayes, Macros Japan, Tokyo, Japan). We measured referral frequencies by referrals to emergency departments and to other medical facilities. We confirmed the actual numbers of visits to off-island facilities, visits to emergency departments, and hospitalizations using the response letters from referral facilities and/or information that the patients actually visited the referral facilities obtained from patient records.

We compared the incidence rates for visits, referrals, and hospitalizations with those reported in a previous study [11]. The category, “visit to an outpatient office excluding primary care facilities” used in the present study was defined as referral to a hospital-based outpatient clinic or a secondary care clinic (e.g., ophthalmological clinic) off the island. In Japan, there are secondary care clinics that are not hospital-based (i.e., ophthalmologists, otolaryngologists). We compared this category with the category “hospital-based outpatient clinic visits” used in the previous study [11].

To compare the ecology of the island’s medical care with that described in the previous study [11], it was necessary to consider information on the proportion of the population aged 65 years and over, health status (e.g., prevalence of non-communicable diseases and neoplasm), and socioeconomic status. However, that study [11] did not present this information. Therefore, we substituted data for Japan overall from the Japan Ministry of Health, Labour and Welfare [17]/Japanese Statistics Bureau [18] for those comparisons. We calculated the prevalence of non-communicable diseases and neoplasm on Iheya using the actual number of patients visiting the island’s clinic as the numerator and the island’s population as the denominator. We used data for socioeconomic status in Iheya from the Japanese Statistics Bureau [18]/Okinawa Prefecture [19].

### Statistical analysis

We performed descriptive analyses to demonstrate healthcare-seeking behavior per 1000 inhabitants over a 1-month period based upon the actual number of visits. We calculated 95% confidence intervals (CI) for event rates obeying either a normal distribution (≥10 events) or a Poisson distribution (<10 events).

## Results

In total, there were 5682 patient visits to the clinic over the 1-year study period (2790 men, 2892 women). Of these, 2615 (1151 men, 1464 women) were aged ≥65 years, 2205 (1209 men, 996 women) were aged 15–64 years and 862 (430 men, 432 women) were aged <15 years. There were 290 referrals (147 men, 143 women), 64 (33 men, 31 women) of which were referrals to emergency departments. Of the 64 referred patients, 57 (30 men, 27 women) were admitted to hospitals. Of the 290 referrals, there were 224 referrals to hospital-based outpatient clinics and 66 referrals to secondary care clinics. Of those referred to hospital-based outpatient clinics and secondary care clinics without emergency, only one patient was admitted to hospital.

We calculated the number of visits and referrals for 1 month per 1000 inhabitants. The results showed that 360.4 (95% CI: 351.0 − 369.7) inhabitants visited the clinic. Of these visitors, 18.4 (95% CI: 16.3 − 20.5) were referred to off-island medical facilities. These referrals included 4.1 (95% CI: 3.1 − 5.1) referrals to emergency departments and 3.6 (95% CI: 2.6 − 4.6) hospitalizations. The referral rate to university hospitals was less than 1, and no patients were admitted to a university hospital during the study period. Table 1 shows the overall and category-specific incidence rates by age and sex.

**Table 1:** Healthcare-seeking behavior for 1 month per 1000 inhabitants

	Primary care clinic visits (95% CI)	Referrals to other medical facilities (95% CI)	Referrals to emergency department (95% CI)	Hospitalization (95% CI)
Overall	360.4 (351.0–369.7)	18.4 (16.3–20.5)	4.1 (3.1–5.1)	3.6 (2.6–4.6)
Age (years)				
< 15	298.1 (278.2–318.0)	5.5 (2.8–8.2)	1.0 (−0.1–2.2)	0.7 (−0.3–1.7)
15–64	247.6 (237.3–258.0)	11.0 (8.8–13.2)	2.2 (1.3–3.2)	1.7 (0.8–2.5)
≥ 65	658.4 (633.1–683.6)	44.3 (37.8–50.9)	10.3 (7.2–13.5)	10.3 (7.2–13.5)
Sex				
Men	342.4 (329.7–355.1)	18.0 (15.1–21.0)	4.1 (2.7–5.4)	3.7 (2.4–5.0)
Women	379.3 (365.4–393.1)	18.8 (15.7–21.8)	4.1 (2.6–5.5)	3.5 (2.2–4.9)

*CI* confidence interval

Table 2 shows the age-adjusted mortality rate and standardized mortality ratio of the island compared with Okinawa Prefecture [20].

**Table 2:** Comparison of the age-adjusted mortality and the standardized mortality ratio between Iheya island and Okinawa Prefecture [20]

2003–2012	Age-adjusted mortality	Standardized mortality ratio
Men on Okinawa : Iheya	636.14 : 610.71	100 : 105.84
Women on Okinawa : Iheya	533.08 : 422.81	100 : 94.74

**Fig. 2:**
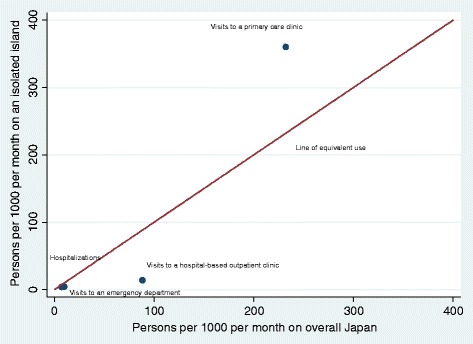
Comparison of medical care services between the present study and a previous study targeting Japan [11]

A comparison between the healthcare-seeking behavior demonstrated in the present study and that of a previous study targeting Japan [11] is shown in Table 3 and Fig. 2. The present study found a higher incidence of visits to the primary care clinic than the previous study [11] In contrast, the incidence rates of visits to hospital-based outpatient clinics, emergency departments, and hospitalizations in the present study were lower than those previously reported [11].

**Table 3:** Comparison of healthcare-seeking behavior (/1000 inhabitants/month)

	Fukui et al. 2005 [11] Japan overall (95% CI)	Iheya island, Japan (95% CI)
Visits to a primary care clinic^a^	232.3 (218.4–246.8)	360.4 (351.0–369.7)
Visits to an outpatient office (excluding primary care facilities)	88.3^b^ (79.1–98.2)	18.4^c^ (16.3–20.5) 14.2^b^ (12.3–16.1) 4.2^d^ (3.2–5.2)
Visits to an emergency department	9.8 (6.8–13.6)	4.1 (3.1–5.1)
Hospitalization	7.2 (4.7–10.6)	3.6 (2.6–4.6)

*CI* confidence interval

^a^Since the category, “Visits to a primary care office”, used in the Fukui’s study was equivalent to “Visit to a primary care clinic” in the present study, we unified the expression to “Visit to a primary care clinic”

^b^Data for people visiting hospital-based outpatient clinics

^c^Data for people visited to hospital-based outpatient clinics and secondary care clinics (e.g., ophthalmological clinics)

^d^Data for people visited to secondary care clinics

As factors such as the proportion of the aging population, the prevalence of non-communicable diseases, and socioeconomic status are likely to be associated with healthcare-seeking behavior, we compared these factors between the present study (conducted in 2013–2014) and the 2003 nationwide survey. We found that 25.1% of Iheya’s population was aged 65 years and over (2013) versus 19.0% in Japan overall (2003) [17]. The prevalence of non-communicable diseases and neoplasm were: hypertension, 22.1% (Iheya, 2013) vs. 5.4% (Japan overall, 2002 [17]); diabetes mellitus, 4.1% (Iheya, 2013) vs. 1.7% (Japan overall, 2002 [17]); and neoplasm, 3.0% (Iheya, 2013) vs. 1.0% (Japan overall, 2002 [17]). In terms of socioeconomic status, the 2013 per capita income in Iheya was 1,735,000 yen [19] compared with 3,086,000 yen [18] for Japan in 2003. The unemployment rate in Iheya in 2010 was 4.1% compared with 2.5% for Japan in 2005 [18]. The proportion of high school graduates in Iheya (2010) was 32.6% compared with 35.5% for Japan in 2000 [18]. In addition, the proportion of university graduates in Iheya (2010) was 5.3% compared with 11.5% in Japan in 2000 [18].

## Discussion

This study described the ecology of medical care by measuring the healthcare-seeking behavior of inhabitants of an isolated island with limited access to advanced care. The descriptive analyses showed that the incidence rates for visits to hospital-based outpatient clinics, visits to emergency departments, and hospitalizations were lower than those reported in a previous Japanese study [11]. However, more inhabitants visited the island’s primary care clinic.

Our finding of more visits to a primary care clinic may be explained by the high prevalence of non-communicable diseases and neoplasm, accessibility of primary care, the lack of access to secondary medical facilities, the lack of other medical resources on the island (i.e., pharmacy), and the increased rate of aging in the island’s population. The low socioeconomic status of the island’s population might also be associated with more clinic visits, as poorer social and economic circumstances are correlated with serious diseases and premature death [21]. Despite the high prevalence of non-communicable diseases, the incidence rates of visits to hospital-based outpatient clinics, emergency departments, and hospitalizations were lower than the rates reported in the previous nationwide survey. This suggests that several dimensions of primary care [12, 22] are likely to play important roles in this geographical setting. In particular, this result implies that if the primary health care system in Japan was a “gatekeeping system” rather than a “free access system,” primary care might play an important role in reducing visits to secondary care, emergency department visits, and hospitalizations. Another possibility is that the low socioeconomic status might have also inhibited visits to off-island medical facilities. However, socioeconomic status is likely not responsible for the all discrepancy of visit to hospital-based outpatient clinics between the present study (14.2/1000 inhabitants/month) and the previous nationwide study (88.3) due to universal health insurance coverage.

Although the previous study in Japan [11] and our study used different methods, selection bias was minimized in both studies. The previous study [11] used a population weighted random sample and achieved a high response rate (health diaries were sent to 1464 households; 1359 households/3658 people returned the diaries, and the data were complete for 3477 people), and our study covered all of Iheya’s inhabitants.

Although we did not measure the number of visits to off-island primary care facilities without referrals from the island’s clinic, it was likely to be small as the island is geographically isolated and access to the main island of Okinawa is limited. It is difficult to conclude whether or not the primary care function in the present study was efficient; however, the clinic carried out healthcare of appropriate quality, including valid triage functions for the following reasons. First, the island’s the standardized mortality ratio, based on Okinawa Prefecture was almost 100 for both males and females [20]. Second, the island’s annual health care cost per capita (241,408 Japanese yen) was less than that of Okinawa Prefecture (251,282 Japanese yen) [23]. Finally, we found that there were 4.9 referrals to specialists per 100 consultations, which is almost the same as in the United States (5.1) and the United Kingdom (4.7) [24].

### Limitations

Although we expected the majority of the island’s inhabitants to use the primary care clinic on the island, it may be possible that some inhabitants regularly visited off-island medical facilities or visited emergency departments without a referral from the island’s primary care clinic. Moreover, some cases, such as patients with terminal cancer or dialysis, might have moved off the island because of the difficulties associated with regularly visiting medical facilities on the main island of Okinawa. Therefore, the present study might have underestimated the number of visits and referrals.

This study was performed with only one physician in one clinic; therefore, the results might depend on the ability of that physician. Furthermore, comparisons of the standardized mortality ratio and healthcare costs between the present study and Okinawa Prefecture might not be sufficient to assess the quality of medical care.

## Conclusion

This study described the ecology of medical care with limited access to advanced care in Japan. Despite many inhabitants visiting the primary care clinic on their geographically isolated island, the rates of visits to hospital-based outpatient clinics, visits to emergency departments, and hospitalizations were lower than those previously reported in Japan. The present study explored a model for estimating the healthcare-seeking behavior on an isolated island where access to secondary care is limited. This result implies that if Japan’s primary healthcare system was a “gatekeeping system” rather than a “free access system,” primary care might play an important role in reducing visits to secondary care, emergency department visits, and hospitalizations. To improve the health status of the island’s inhabitants, it is important for the primary care clinic to be involved in disease prevention. In addition, we plan to repeat this study at 10- and 20-year intervals to reexamine the role of primary care.

## References

[1] White KL, Williams TF, Greenberg BG (1961). The ecology of medical care. N Engl J Med.

[2] Dovey S, Weitzman M, Fryer G, Green L, Yawn B, Lanier D (2003). The ecology of medical care for children in the United States. Pediatrics.

[3] White KL (1997). The ecology of medical care: origins and implications for population-based healthcare research. Health Serv Res.

[4] Thacker SB, Greene SB, Salber EJ (1977). Hospitalizations in a southern rural community: an application of the ‘ecology model’. Int J Epidemiol.

[5] McWhinney IR (1981). An introduction to family medicine.

[6] Godwin M, Grzybowski SC, Stewart M, Labrecque M, Grava-Gubins A, Katz A (1999). Need for an institute of primary care research within the Canadian institutes of health research. Can Fam Physician.

[7] Fryer GE, Green LA, Dovey SM, Yawn BP, Phillips RL, Lanier D (2003). Variation in the ecology of medical care. Ann Fam Med.

[8] Knox PL (1978). The intraurban ecology of primary medical care: patterns of accessibility and their policy implications. Environ Plan A.

[9] Green LA, Fryer GE, Yawn BP, Lanier D, Dovey SM (2001). The ecology of medical care revisited. N Engl J Med.

[10] Johansen ME, Kircher SM, Huerta TR (2016). Reexamining the ecology of medical care. New Engl J Med.

[11] Fukui T, Rhaman M, Takahashi O, Saito M, Shimbo T, Endo H (2005). The ecology of medical care in Japan. JMAJ.

[12] Kringos DS, Wienke GWB, Hutchinson A, van der Zee J, Groenewegen PP (2010). The breadth of primary care: a systematic literature review of its core dimensions. BMC Health Serv Res.

[13] Tamiya N, Noguchi H, Nishi A, Reich MR, Ikegami N, Hashimoto H (2011). Population ageing and wellbeing: lessons from Japan’s long-term care insurance policy. Lancet.

[14] Ministry of Health, Labour and Welfare, Japan. The report on the role of specialists. 2013. http://www.mhlw.go.jp/stf/shingi/2r985200000300ju-att/2r985200000300lb.pdf. (in Japanese) Accessed 10 June 2014.

[15] Motomura K (2012). Reflective practice and situated learning in remote medicine. J Japan Prim Care Assoc.

[16] e-stat, Portal Site of Official Statistics of Japan. Demographic statistics in Japan. 2013. http://www.e-stat.go.jp/SG1/estat/GL02020101.do?method=extendTclass&refTarget=toukeihyo&listFormat=hierarchy&statCode=00200241&tstatCode=&tclass1=&tclass2=&tclass3=&tclass4=&tclass5=. (in Japanese) Accessed 10 June 2014.

[17] Ministry of Health, Labour and Welfare, Japan. Estimated number of patients receiving medical treatment for selected diseases. 2002. .http://www.mhlw.go.jp/toukei/saikin/hw/kanja/02/5.html. (in Japanese) Accessed 10 Aug 2016.

[18] e-stat, Portal Site of Official Statistics of Japan. Regional statistics database. https://www.e-stat.go.jp/SG1/chiiki/CommunityProfileTopDispatchAction.do?code=2. (in Japanese) Accessed 10 Aug 2016.

[19] Okinawa Prefecture. Municipal income. http://www.pref.okinawa.jp/toukeika/ctv/2016/H25_okinawakenshithousonminsyotoku.pdf. (in Japanese) Accessed 10 Aug 2016.

[20] Division of Health and Welfare, Okinawa Prefecture. Municipal standardized mortality ratio. http://www.kenko-okinawa21.jp/090-docs/2015121400699/. (in Japanese) Accessed 8 Jan 2017.

[21] World Health Organization. Social determinant of health SOLID FACTS 2nd edition. 2003. http://www.euro.who.int/__data/assets/pdf_file/0005/98438/e81384.pdf. Accessed 12 Aug 2016.

[22] Kringos DS, Boerma WGW, Hutchinson A, Saltman RB. Building primary care in a changing Europe. Kringos DS, editor. World Health Organization, European Observatory on Health Systems and Policies; 2015. http://www.euro.who.int/__data/assets/pdf_file/0018/271170/BuildingPrimaryCareChangingEurope.pdf. 29035488

[23] Iheya Village. Second phase of specific screening implementation plan. 2010. http://www.vill.iheya.okinawa.jp/UserFiles/File/ju-min/kenkou/keikaku/5.P14-29.pdf. (in Japanese) Accessed 26 June 2014.

[24] Forrest CB (2003). Primary care gatekeeping and referrals: effective filter or failed experiment?. BMJ.

